# Thermostability Improvement of L-Asparaginase from *Acinetobacter soli* via Consensus-Designed Cysteine Residue Substitution

**DOI:** 10.3390/molecules27196670

**Published:** 2022-10-07

**Authors:** Linshu Jiao, Huibing Chi, Bingjie Xia, Zhaoxin Lu, Xiaomei Bie, Haizhen Zhao, Fengxia Lu, Meirong Chen

**Affiliations:** College of Food Science and Technology, Nanjing Agricultural University, Nanjing 210095, China

**Keywords:** L-asparaginase, *Acinetobacter soli*, thermostability, consensus design, cysteine substitution, acrylamide inhibition

## Abstract

To extend the application range of L-asparaginase in food pre-processing, the thermostability improvement of the enzyme is essential. Herein, two non-conserved cysteine residues with easily oxidized free sulfhydryl groups, Cys8 and Cys283, of *Acinetobacter soli* L-asparaginase (AsA) were screened out via consensus design. After saturation mutagenesis and combinatorial mutation, the mutant C8Y/C283Q with highly improved thermostability was obtained with a half-life of 361.6 min at 40 °C, an over 34-fold increase compared with that of the wild-type. Its melting temperature (*T*_m_) value reaches 62.3 °C, which is 7.1 °C higher than that of the wild-type. Molecular dynamics simulation and structure analysis revealed the formation of new hydrogen bonds of Gln283 and the aromatic interaction of Tyr8 formed with adjacent residues, resulting in enhanced thermostability. The improvement in the thermostability of L-asparaginase could efficiently enhance its effect on acrylamide inhibition; the contents of acrylamide in potato chips were efficiently reduced by 86.50% after a mutant C8Y/C283Q treatment, which was significantly higher than the 59.05% reduction after the AsA wild-type treatment. In addition, the investigation of the mechanism behind the enhanced thermostability of AsA could further direct the modification of L-asparaginases for expanding their clinical and industrial applications.

## 1. Introduction

L-asparaginase (EC3.5.1.1) is a kind of amidohydrolase that can catalyze the deamination of L-asparagine to L-aspartate [1]. L-asparaginase is widely used in the clinical treatment of acute lymphoblastic leukemia and other leukemia by depleting L-asparagine in the plasma, thus inhibiting tumor cell growth [2,3]. L-asparaginase can also be applied in the food industry to reduce the formation of acrylamide [4], a potential carcinogen formed via the Maillard reaction from reducing sugar and L-asparagine in various food products processed at high temperatures such as coffee, bread, French fries, and cookies [5,6]. L-asparaginase can effectively reduce the content of acrylamide in food processing by degrading L-asparagine, the precursor of acrylamide, to minimize the carcinogenic risk of foods [7,8,9].

L-asparaginases are usually used in food pre-processing, the complex and diverse reaction environment of food pretreatment makes the thermostability of L-asparaginase essential, meanwhile, L-asparaginase treatment combined with blanching showed better acrylamide inhibition [10]. To meet this requirement, extensive studies were carried out to obtain thermostable L-asparaginases. The molecular modification of known enzymes is an important alternative means of developing thermostable L-asparaginases. Traditional methods, particularly directed evolution, were employed to introduce considerable random mutations of the engineered proteins and to obtain mutants with excellent properties. However, as an engineering strategy emulating natural evolution processes, directed evolution is very dependent on the quantities and qualities of constructed mutation libraries [11,12]. It is usually difficult to obtain desirable mutants due to the cost and time-consuming screening processes. Thus, exploring efficient and suitable screening methods for variant selection remains imperative. In recent years, the computational engineering design of enzymes has been developed as an alternative means for thermostability improvement, where the screening works are reduced compared with directed evolution. The computational engineering design includes two complementary approaches based on the available information on proteins. Structure-based design requires the structure information of proteins to locate the flexible regions by flexibility/rigidity analysis, such as B-factor analysis [13], molecular dynamics (MD) simulations [14], and Rosetta design [15].

For enzymes whose structures remain unsolved and have low structural homology to the resolved structures in the database, a sequence-based design could be a promising alternative approach, which is based on the available database of the homologous sequences with known properties, and mutation sites are selected according to the conservation of the residues [16]. For example, consensus design could generate consensus residues at given positions through multiple sequence alignment (MSA) of available sequences of the family, which then directs the substitution of the residues of the target protein into the corresponding conserved ones [17,18]. The above two approaches were utilized in combination in many cases. Li et al. [19] sorted out the specific residues and regions that may affect the thermostability of the enzymes by sequence and structure alignments of thermophilic and non-thermophilic L-asparaginases. They eventually determined the two key residues responsible for the thermostability of thermophilic and non-thermophilic L-asparaginases through enzymatic characterization and MD simulation of the mutants.

In our previous work, a novel L-asparaginase was cloned from the *Acinetobacter soli* Y-3 (AsA) and heterologously expressed in *Escherichia coli* BL21 (DE3), which had high catalytic activity and the ability to inhibit acrylamide formation during the processing of potato chips; however, the recombinant AsA showed poor thermostability with a half-life at 40 °C of 4.1 min, and DTT and β-mercaptoethanol had greatly enhanced AsA activity by 383% and 170%, respectively [20]. Considering that these two thiol-reducing agents can prevent the oxidation of free sulfhydryl groups and there is no potential for inner molecular disulfide bond formation in AsA, we speculate that there are unstable cysteine residues and mismatched intermolecular disulfide bonds in AsA which caused its poor thermostability.

In this study, a mutation strategy of cysteine residue substitution based on the consensus design was carried out to enhance the thermostability of AsA. Cysteine residues of AsA that were not conserved in the evolution were selected and mutated. Positive mutants with significantly improved thermostability were obtained. To obtain insights into the mechanism of the improved thermostability, homology modeling and MD simulation were also performed with AsA mutants. As a result, we concluded that the additional hydrogen bonds and conjugation force introduced by mutation greatly contribute to the thermostability of the mutants.

## 2. Results

### 2.1. Cys8 and Cys283 with Low Conservation Selected for Mutagenesis to Enhance the Thermostability of AsA

**Figure 1:**
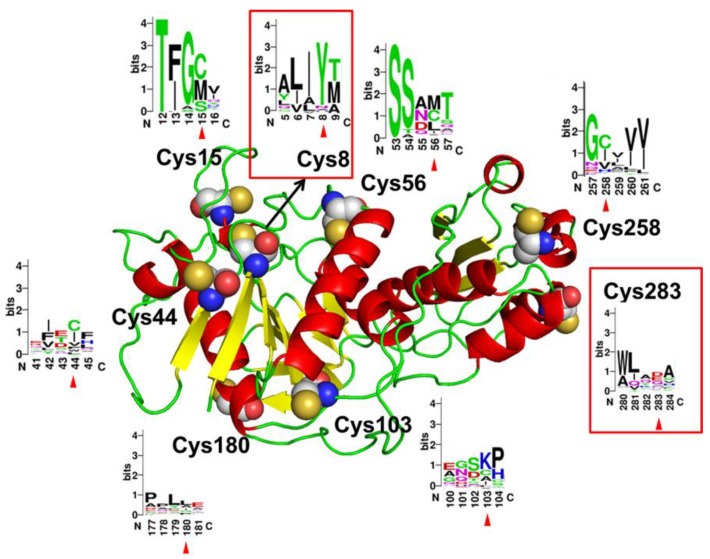
Structure model of AsA monomer. Eight cysteine residues are shown in spheres and the relative frequencies of each amino acid at the corresponding sites are shown with WebLogo 3.

A mutation strategy of cysteine residue substitution based on the consensus design was carried out. According to the prediction result generated by PredictProtein, no disulfide bond was found inside the AsA molecule. Moreover, with the structure model of AsA (Figure 1), we found seven cysteine residues located on the surface of the enzyme and one located on the interface between the two subunits of the AsA, which means that they all have the possibility of being oxidized and form unexpected intermolecular disulfide bonds with other AsA molecules, resulting in the poor thermostability of AsA. To determine the conservation of the eight cysteine residues, a sequence-based consensus design was employed by the Consensus Finder Web server with 308 homologous sequence inputs. The generated consensus sequence is shown in Supplemental Figure S1. Meanwhile, the frequencies of the corresponding sites of cysteine residues were analyzed and shown using WebLogo 3 (Figure 1). The structure model of AsA was obtained using SWISS-MODEL online server with *Thermococcus kodakarensis* L-asparaginase (PDB ID: 5OT0, 24.44% identity, 2.18 Å resolution) as a template and the structure quality analysis of the model is shown in Table S1. In total, 92.8% of residues were located in the most favored regions and 7.2% of the residues were located in additionally allowed regions. Eight cysteine residues in AsA, Cys15, Cys44, and Cys258 were conserved throughout the evolution. Cys56, Cys103, and Cys180 emerged at frequencies of 28%, 15%, and 6%, respectively; Cys8 and Cys283 emerged at a frequency lower than 1% and were selected for saturated mutagenesis.

### 2.2. Thermostability of the AsA Variants

Saturation mutations were carried out at Cys8 and Cys283 and mutants still retained catalytic activities after thermal treatment (Table 1). However, after half-life determination, only three mutants were present at residue Cys8 (C8Y, C8F, C8W). One mutant at residue Cys283 (C283Q) exhibited an extended half-life at 45 °C. Double mutants were also constructed to evaluate the contribution of the two site mutations. The results of the SDS-PAGE analysis of the mutants are shown in Supplemental Figure S2.

**Figure 2:**
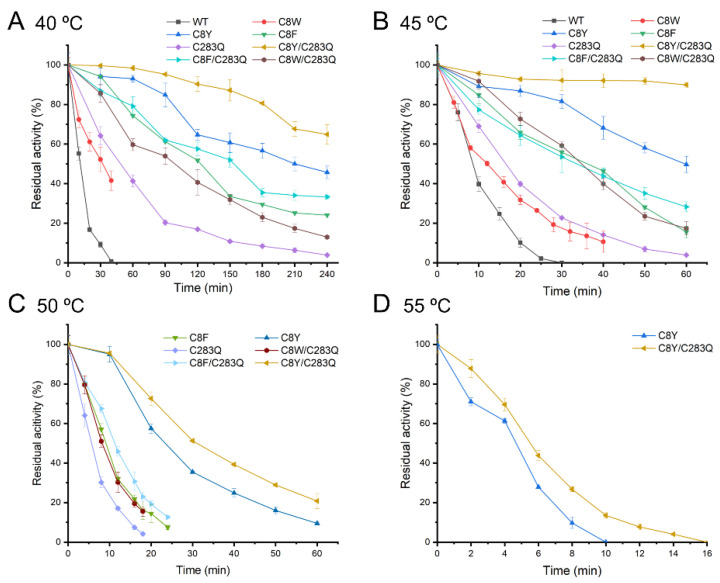
Thermostability of AsA wild-type and mutants under 40 °C (**A**), 45 °C (**B**), 50 °C (**C**), and 55 °C (**D**). Data are expressed as mean values (±SD) of three independent experiments.

The thermostability of the mutants was determined at 40, 45, 50, and 55 °C. The residual activities of the mutants incubated at different temperatures for a period were graphed as shown in Figure 2, and the corresponding half-life was calculated (Table 2). At 40 and 45 °C, the half-lives of the AsA wild-type were 9.1 and 4.1 min, respectively. Among the mutants, the mutant C8W had the least improved thermostability with a half-life of 25.2 min at 40 °C and 12.3 min at 45 °C. The mutant C8F exhibited better thermostability with 11.3- and 5.8-fold higher half-lives at 40 and 45 °C than the wild-type. Most importantly, the mutant C8Y had the highest thermostability. The half-lives at 40 and 45 °C were 15.7- and 13.1-fold higher than those of the wild-type, respectively. In particular, the mutant C8Y could retain half of its activity even after incubation at 40 °C for 4 h. The mutant C283Q also had moderate thermostability, with a half-life of 52.5 and 12.5 min at 40 and 45 °C, respectively. As Cys8 and Cys283 negatively contribute to the thermostability of AsA, the double mutants, C8Y/C283Q, C8F/C283Q, and C8W/C283Q, were also evaluated. The combined mutation has a superimposing effect in terms of the enzyme thermostability, as the double mutants all showed better thermostability than single-point mutants. Especially, the mutant C8Y/C283Q had a half-life of 361.6 min at 40 °C, which was nearly 40-fold longer than that of the wild-type and two-fold longer than the mutant C8Y. The mutant C8Y/C283Q also had a good performance at 45 °C as it could maintain about 90% residual activity after 1 h incubation. However, it deactivated quickly when the thermal treatment lasted more than 1 h (Supplemental Figure S3), with a half-life of 68.0 min at 45 °C (Table 2).

Due to the poor thermostability of the AsA wild-type, its half-life at 50 and 55 °C could not be determined. At 50 °C, the mutant C8Y showed a half-life of 16.84 min, whereas the mutant C8Y/C283Q had a half-life of 25.21 min. The half-lives of mutants C8F, C283Q, C8F/C283Q, and C8W/C283Q ranged from 3.5 to 8 min. At 55 °C, only the half-life of mutants C8Y and C8Y/C283Q was measurable, which were both less than 3 min.

As one of the important indicators to characterize the thermostability of enzymes, the *T*_m_ of the mutants was measured by differential scanning fluorometry. All mutants showed higher *T*_m_ than the wild-type (Table 2). Compared with the half-life analysis, the mutant C8Y/C283Q showed the highest *T*_m_ value of 62.3 °C, which was 7.1 °C higher than that of the wild-type. The mutant C8W/C283Q also showed obvious improvement with a *T*_m_ value of 60.9 °C, 5.6 °C higher than that of the wild-type.

### 2.3. Optimum Temperature of AsA Variants

**Figure 3:**
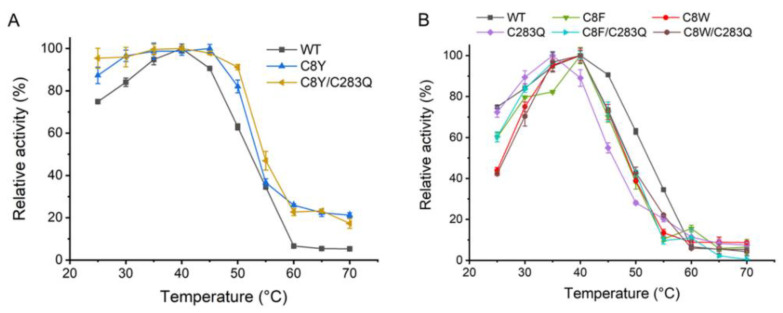
Optimum temperature of mutants C8Y and C8Y/C283Q (**A**) and optimum temperature of mutants C8F, C8W, C283Q, C8F/C283Q, and C8W/C283Q (**B**). The activity at optimum temperature of each enzyme was set as 100%, respectively.

The effects of temperature on the mutants’ catalytic activities were also determined and the activity at the optimum temperature of each enzyme was set as 100%, respectively. The results showed that mutants C8Y and C8Y/C283Q could obviously tolerate a broader range of temperature compared with the wild-type (Figure 3A). The AsA wild-type was active between 25 and 55 °C with the optimum temperature of 40 °C, retaining 60% relative activity at 50 °C and less than 10% relative activity at 60 °C. By contrast, the mutant C8Y retained more than 80% relative activity between 25 and 50 °C and 95% relative activity at 35–45 °C. Notably, the mutant C8Y/C283Q showed greater than 90% relative activity between 25 and 50 °C and 95% relative activity at 30–45 °C. Even at 70 °C where the wild-type had only about 5% relative activity, mutants C8Y and C8Y/C283Q still had around 20% relative activity left. The effects of temperature on the catalytic activities of mutants C8F, C8W, C283Q, C8F/C283Q, and C8W/C283Q are shown in Figure 3B. Mutants C8F, C8W, C8F/C283Q, and C8W/C283Q shared similar optimum temperature and reaction temperature regions with the wild-type at 40 °C. Moreover, the optimum temperature of mutant C283Q was 35 °C, which was lower than that of the wild-type, and its reaction temperature range was narrow correspondingly.

### 2.4. Kinetic Parameters of the AsA Variants

To determine the impact of these two mutations on the catalytic function of the enzyme, the specific activities and kinetic parameters of AsA and its mutants were determined with L-Asn as the substrate (Table 3). Compared with the AsA wild-type, mutants C8Y, C8F, C283Q, and C8Y/C283Q showed enhanced specific activities from 13.2 to 56.1%. However, the kinetic parameters of these mutants were not improved as they showed decreased substrate affinities (*K*m values larger than 10 mM) and slightly lower catalytic efficiencies (Table 3). The mutants C8W, C8F/C283Q, and C8W/C283Q showed decreased specific activities. Among them, the mutant C8W, with the least thermostability improvement, showed the lowest specific activity and *k*cat, whereas it had the highest substrate affinity. Notably, mutants C8F/C283Q and C8W/C283Q had a remarkably increased *k*cat/*K*m of 60.9 and 61.0 s^−1^·mM^−1^, respectively, compared with the wild-type with a *k*cat/*K*m of 52.0 s^−1^·mM^−1^. This finding suggests that not only the thermostability but also the catalytic efficiency of the mutants C8F/C283Q and C8W/C283Q were greatly enhanced.

### 2.5. Model Analysis and MD Simulation of the AsA Variants

**Figure 4:**
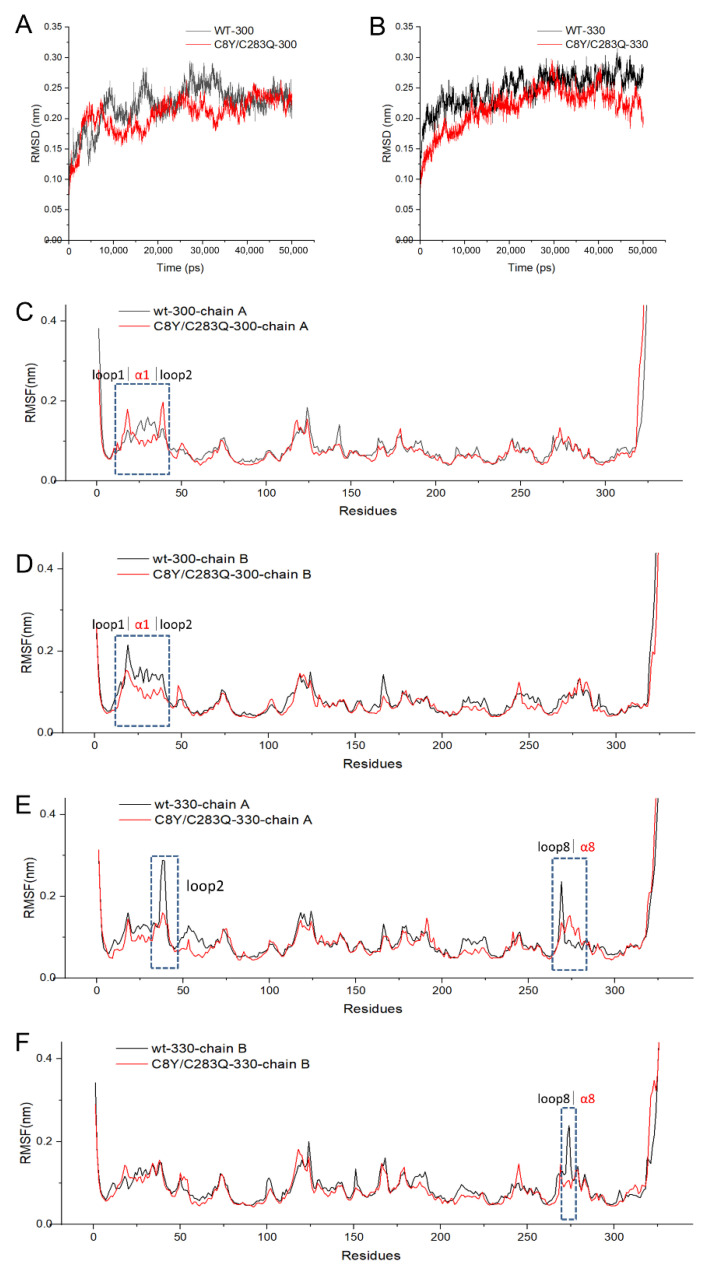
RMSD and RMSF values of AsA and mutant C8Y/C283Q over time during MD simulation. (**A**) RMSD values of AsA and mutant C8Y/C283Q simulated at 300 K. (**B**) RMSD values of AsA and mutant C8Y/C283Q simulated at 330 K. (**C**) RMSF values of chain A in AsA and mutant C8Y/C283Q simulated at 300 K. (**D**) RMSF values of chain B in AsA and mutant C8Y/C283Q simulated at 300 K. (**E**) RMSF values of chain A in AsA and mutant C8Y/C283Q simulated at 300 K. (**F**) RMSF values of chain B in AsA and mutant C8Y/C283Q simulated at 300 K. The parts marked by the dotted line were the areas with large difference between wild-type and mutant.

To obtain insights into the structural contribution of the two cysteine substitutions to the thermostability of AsA, the structure models of mutant C8Y/C283Q were constructed based on the structure model of wild-type and the structure quality analysis of the model is shown in Table S1. In total, 92.8% of residues were located in the most favored regions and 7.2% of residues were located in additionally allowed regions. The MD simulations were performed by Gromacs. The RMSD values of the backbone of enzymes after 10 ns simulation are shown in Figure 4. The RMSD values of the wild-type reached 0.2 nm after 1.8 ns of simulation, stabilizing at around 0.25 nm after 10 ns of simulation. The RMSD values of the mutant C8Y/C283Q were stabilized at 0.20–0.23 nm during the simulation. When the simulation temperature increased to 330 K, the RMSD value of the wild-type reached 0.23 nm after 4 ns, stabilizing at around 0.27 nm. The RMSD values of mutant C8Y/C283Q were around 0.22 nm, still lower than that of the wild-type, suggesting the reduced flexibility of the mutant (Figure 4B).

In addition to the RMSD values, the number of hydrogen bonds, the radius of gyration (Rg) and the solvent-accessible surface area (SASA) of the protein during the simulation process can also reflect the stability (Table 4).

Hydrogen bonds are one of the most important forces affecting protein stability, the more the bonds number, the tighter the internal binding of proteins. The numbers of intramolecular hydrogen bonds of AsA wild-type and are waere shown in Table 4. When simulated at 300 K, the average intramolecular hydrogen bonds number of mutant C8Y/C283Q and wild-type were 457.1 and 489.3, the values were 461.8 and 494.8 at 330 K. The bond numbers of mutant C8Y/C283Q were always larger than those of wild-type, which was consistent with the results of the thermostability determination. Rg value is related to the tightness of the protein structure, the outward expansion of the protein and the loose structure. At 300 K, the average Rg values of the AsA wild-type and mutant C8Y/C283Q were 3.1 nm and 2.7 nm, respectively, indicating the more compact overall structure of the protein after mutation. At 330 K, the average Rg values of the AsA wild-type decreased to 2.6 nm, and that of mutant C8Y/C283Q had little change. The SASA value is an important parameter to describe the protein hydrophobicity. The SASA values of the AsA wild-type and mutant C8Y/C283Q were 263.4 and 259.5 Å^2^ at 300 K, and 260.0 and 256.8 Å^2^ at 330 K. The lower SASA values of the mutant indicated it had stronger hydrophobicity and a tighter structure than the wild-type.

RMSF values reflect the flexibility of each residue during simulations, which is a significant parameter indicating the thermostability of proteins. As shown in Figure 4C,D, when simulated at 300 K, both chains A and B of mutant C8Y/C283Q and wild-type showed little difference in RMSF values, except for the region of loop1, α1 and loop2. In chain A, the RMSF values of the mutant C8Y/C283Q at loop1 and loop2 were higher than those of the wild-type, and the RMSF values of the mutant C8Y/C283Q at α1 were lower than those of the wild-type. In chain B, the RMSF values of the mutant C8Y/C283Q at loop1, α1 and loop2 were lower than those of the wild-type. When simulated at 330 K (Figure 4E,F), some regions in chain A of the mutant C8Y/C283Q, such as loop1 and loop8, showed obviously reduced RMSF values compared to the wild-type, while the RMSF values of α8 nearing loop8 were higher than those of the wild-type. In chain B, the only difference between the wild-type and mutant was the RMSF values at loop8, indicating the flexibility change of this region. The mutation of residues Cys8 and Cys283 influenced the flexibility of regions nearby rather than the whole molecule.

**Figure 5:**
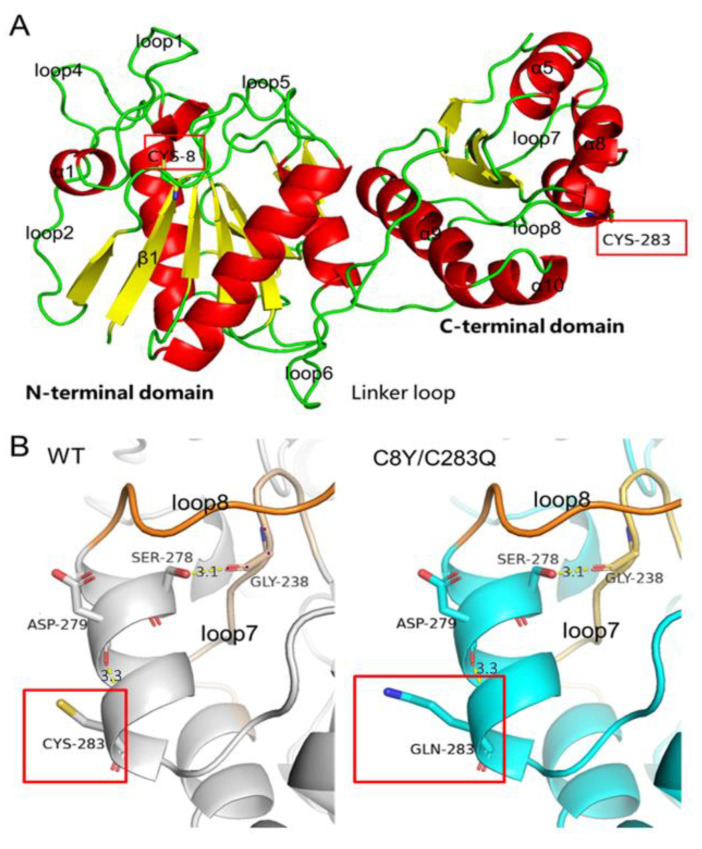
Alteration in the model structures of AsA and mutant C8Y/C283Q. (**A**) Location of Cys8 and Cys283 on AsA monomer. (**B**) Structure comparison around residue Cys283 of the wild-type and the mutated residue (Gln283) of mutant C8Y/C283Q.

The structure model analysis of the mutant C8Y/C283Q and wild-type was further conducted to clarify the thermostability-enhancing mechanism of AsA. The main changing region loop8 (residues 265–275) was next to the α8 where the mutation C283Q was located (Figure 5A). In the structure model of the AsA, no cysteine residue was present near the residue Cys283 for the disulfide bond formation, which means that the free –SH group of Cys283 pointing toward the solute outside the protein was vulnerable to oxidation by various factors (Figure 5B).

The only residue in the protein that may form a hydrogen bond with residue Cys283 was residue Asp279, and the predicted intramolecular hydrogen bond formed by residue Cys283 was 1.8 at 300 K and 1.6 at 330 K, and the predicted hydrogen bond numbers between Cys283 and other molecules were 2.0 at 300 K and 1.8 at 330 K (Table 5). After substitution, the residue Gln283 could also form a hydrogen bond with residue Asp279 (Figure 5C), and the oxygen and amino group on the side chain of glutamine offered more opportunity for hydrogen bond formation as the predicted average hydrogen bond numbers of Gln283 formed with other residues inside the enzyme molecule were 2.320 at 300 K and 2.207 at 330 K, and the predicted hydrogen bond numbers between Gln283 and other molecules were 8.631 at 300 K and 8.308 at 330 K (Table 5). Therefore, we believe that the additional force introduced by the substitution of residue Cys283 made the overall structure stable and decreased the flexibility of its vicinal region.

**Figure 6:**
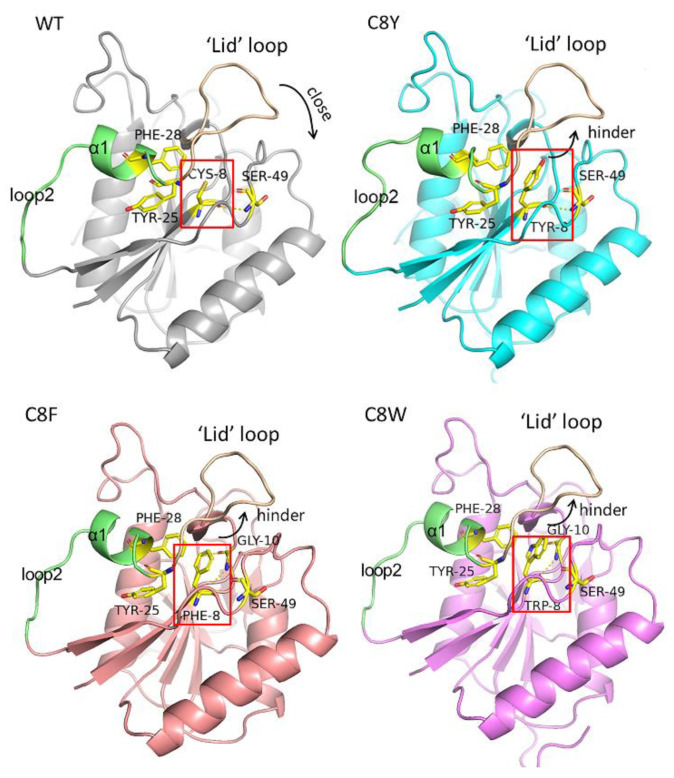
Structures comparison around residue 8 in AsA wild-type and its mutants.

Meanwhile, the RMSF values of loop1 (residues 15–26), α1(residues 27–31) and loop2 (residues 32–40) changed after cysteine substitution. These regions were located next to the helix Cys8 (Figure 5A). This could be reasonable as the single-site mutation can hardly affect the flexibility of the backbone because of the stable hydrogen bond connection between the backbone chains of these β-strands. Residue Cys8 was located on a β-strand of seven-stranded β-sheets in the N-terminal domain, a conserved structure of most L-asparaginases (Figure 6). Moreover, due to the significant position of residue Cys8, the mutation had a great influence on the flexibility of its surrounding structure. In the wild-type, a pair of hydrogen bonds were formed between Cys8 and Ser49. This bond also existed in mutant C8Y/C283Q. The predicted intramolecular hydrogen bond formed by residue Cys8 was 3.4 at 300 K and 3.0 at 330 K, and the predicted hydrogen bond number between Cys8 and other molecules was 0.0 at 300 K and 0.6 at 330 K (Table 5). In mutant C8Y/C283Q, the predicted intramolecular hydrogen bond formed by residue Tyr8 was 5.2 at 300 K and 5.0 at 330 K, and the predicted hydrogen bond number between Cys8 and other molecules was 0.7 at 300 K and 1.2 at 330 K (Table 5).

This indicates that, except for introducing new hydrogen bonds, there could be some additional force after the mutation of Cys8 to Tyr8. After PIC analysis, we found that the substation of residue Cys8 by tyrosine could introduce the aromatic interaction between residue Tyr8 and residues Tyr25 and Phe28 (Figure 6), which stabilized the conformation and improved the thermostability of the mutant. In the mutation of C8F and C8W, a newly formed aromatic interaction was also found (Figure 6). Moreover, residue Cys 8 was located adjacent to the highly flexible ‘lid’ loop on the N-terminal (loop1), whose conformation was related to substrate fixation and catalysis. The residue Cys8 in the wild-type points to the closing direction of this loop, and the loop could close correctly when substrates were combined. However, when replaced by tyrosine residues, their large side chains hindered the closure of the ‘lid’ loop, which affected the substrate catalysis.

### 2.6. Acrylamide Inhibition Analysis of AsA Variants

**Figure 7:**
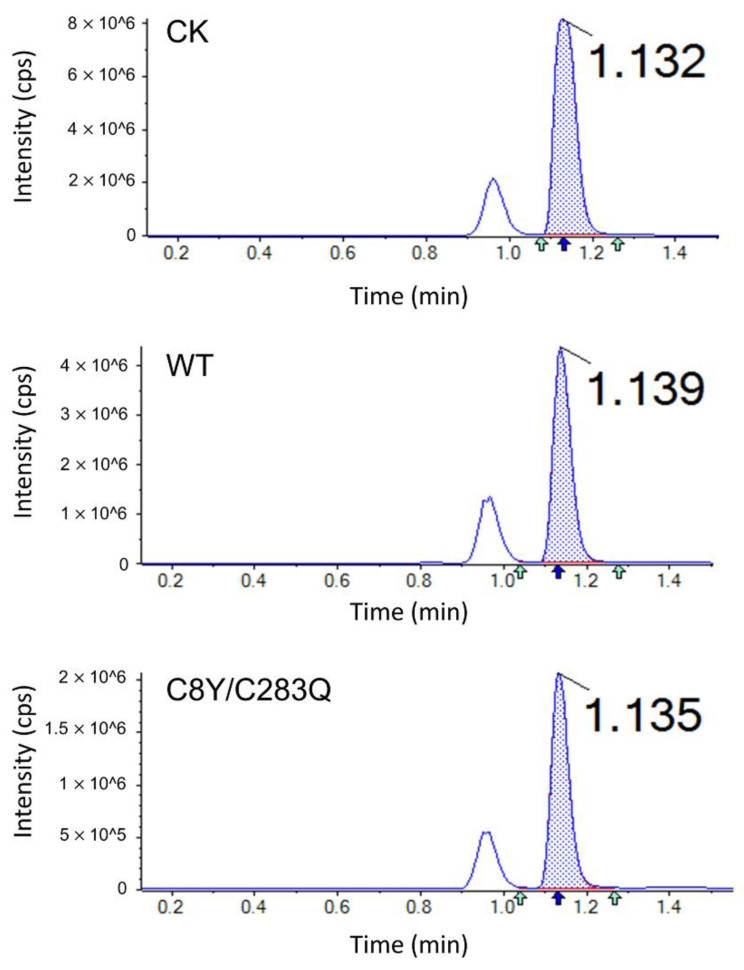
Acrylamide determination by LC–MS after treatment with AsA wild-type and mutant C8Y/C283Q.

The acrylamide determination in potato chips treated with AsA wild-type and mutant C8Y/C283Q was performed using LC–MS, which is shown in Figure 7. The corresponding acrylamide contents and inhibition rates are shown in Table 6. Acrylamide content in the control group was 1.6559 mg/kg; after AsA wild-type treatment, the acrylamide content decreased to 0.6781 mg/kg with an inhibition rate of 59.05%; after mutant C8Y/C283Q treatment, the acrylamide content decreased to 0.2235 mg/kg with an inhibition rate of 86.50%. The inhibition of acrylamide formation was increased by the thermostability improvement of AsA.

## 3. Discussion

Cysteine residues play important roles in protein structure and function. They can stabilize proteins by forming disulfide bonds and their reactive sulfhydryl (-SH) groups enable cysteine residues to form intra- and intermolecular covalent bonds [21]. However, the –SH groups of free cysteine residues can easily be oxidized by metal ions such as Cu^2+^, which negatively affects protein folding [22]. The introduction of a suitable couple of cysteine residues to form a stable covalent bond and the substitution of a cysteine residue with different amino acids was proven effective in eliminating the negative effect of free –SH groups [23,24]. There are many successful cases of cysteine residues substituted by alanine, serine, and valine for increasing the thermostability of enzymes [25,26]. Considering that the structure of AsA is still unknown, constructing new disulfide bonds is not feasible. Therefore, a sequence-based non-conserved cysteine residue substitution was proposed to minimize the oxidization of free cysteine residues on the thermostability of AsA.

Two non-conserved cysteine residues (Cys8 and Cys283) were selected through consensus design. After site-directed saturation mutagenesis, three mutants with aromatic residue substitution on Cys8 were screened out with enhanced thermostability. Especially, the mutant C8Y was characterized by a 15.7-fold increased half-life at 40 °C and a 4.2 °C increased *T*_m_ value. Aromatic interactions can provide van der Waals forces, electrostatic interactions, and hydrophobic interactions. Thus, rational and semi-rational design to introduce new aromatic interactions inside the target protein is one of the effective ways to improve its thermostability [27]. Georis et al. [28] introduced a new Tyr11–Tyr16 aromatic interaction in xylanase, and its optimum temperature and *T*_m_ value increased by 10 °C and 9 °C, respectively. Moreover, Zhang et al. [29] constructed a mutant G414W of *Candida rugosa* lipase 1. The introduced tryptophan residue of the mutant formed a new aromatic interaction with Phe125 and Phe415, leading to increased half-life and optimum temperature. In addition, there was only one positive mutant (C283Q) obtained at position 283, which was partly related to the location of this cysteine residue. Cys283 was located on the surface of the AsA molecule, and its –SH group was easily oxidized [22]. After being substituted by the glutamine residue, the ketone and amino groups on the side chain of Gln283 offered more opportunity for protein–solvent hydrogen bond formation. Adding new intra-protein or protein–solvent hydrogen bonds by altering single amino acids is one of the main strategies to improve the rigidity of the protein structure [30]. For example, although the substitution was not focused on the cysteine residues, Dror et al. [31] constructed a mutant A269T of *Geobacillus stearothermophilus* lipase with an enhanced *T*_m_ value of 3.4 °C, and the altered residue Thr269 formed two new hydrogen bonds with nearby residues. Obviously, these single-point mutations had a significant influence on the thermostability of AsA, which is consistent with many other cases of L-asparaginase modification. *Erwinia chrysanthemi* L-asparaginase with a single-point mutation (D133V) was identified through direct evolution. Compared with the wild-type, its *T*_m_ value increased from 46.6 to 55.8 °C, and this mutation affected the stability of the structure of the enzyme by the optimization of the surface electrostatic potential [32]. Likewise, after the mutagenesis on the same site (D103V), *Bacillus licheniformis* L-asparaginase also exhibited a three-fold enhanced half-life at 37 °C [33]. Furthermore, the double mutants were constructed for better potential properties. Due to the limitation of spatial locations of the mutation sites and different forces introduced, the combined mutants were not always as expected [34]. Double mutants C8Y/C283Q, C8F/C283Q, and C8W/C283Q all exhibited improved thermostability (especially kinetic stability and the thermodynamic stability) than single-point mutants [35]. The most remarkable double mutant C8Y/C283Q had a two-fold higher half-life (40 °C) and increased *T*_m_ value (3 °C) than the mutant C8Y, which indicated the cumulation in the stabilization of the enzyme via the substitution of Cys8 and Cys283.

Improving the stability and activity of the enzymes at the same time is challenging because these two properties result from the flexibility–rigidity trade-off of the proteins [36]. The mechanism of enhanced stability is about the increase in rigidity or the decrease in flexibility and it may have a negative effect on the catalytic activity of the enzymes correspondingly [36,37]. Many cases suggested that the L-asparaginase activity decreased with its stabilization [32], differing from that, mutants C8Y, C8F, C283Q, and C8Y/C283Q showed enhanced thermostability and specific activity simultaneously. The mutant C8Y and its double mutant C8Y/C283Q are both characterized by excellent thermostability, exhibiting 56.12 and 54.90% enhanced specific activity, respectively. Considering the particular location of the mutation sites, we attributed the developed thermostability and catalytic activity to the substitution of residue Cys8 adjacent to the highly flexible loop on the N-terminal. There is a conserved highly flexible loop in L-asparaginases from various resources, which regulates the catalytic function of the enzymes [38]. When there is no ligand binding to the active site, the loop is flexible in an open conformation. However, in the presence of a ligand, the flexible loop will be ordered into a β-hairpin composed of two short β-strands to close the substrate pocket like a “lid,” which pushes the substrate deeper into the pocket [39,40]. Reducing the flexibility of the highly flexible N-terminal loop could improve the specific activity of L-asparaginase. According to the structure analysis, the residue Cys8 in the wild-type points to the closing direction of loop1. After replacing it with tyrosine residues, the introduced aromatic interaction helps to stabilize the structure. However, the large side chain also hinders the closure of the lid, which might be the reason why the catalytic efficiencies of the mutants were not improved effectively.

In this study, the improvement of the thermostability of AsA promoted the inhibition of acrylamide formation in potato chips. Lu et al. [41] applied *Bacillus megaterium* L-asparaginase and its thermostability enhanced mutant to the inhibition of acrylamide in fried potato chips and acrylamide contents in the samples decreased by 78.6 and 88.5%, respectively. After the combined treatment of blanching (80 °C, 1 min) and commercial L-asparaginase from *Aspergillus oryzae* (10,500 ASNU/mL, 40 °C, 15 min), acrylamide content in potato chips decreased from 1.75 to 0.71 mg/kg [10]. The application effect of mutant C8Y/C283Q was better than that of the commercial enzyme, indicating that the improvement of the thermostability effectively enhances its application potential of AsA.

## 4. Materials and Methods

### 4.1. Plasmid, Strains, and Materials

The plasmid pET30a-AsA harboring the L-asparaginase gene from *A. soli* was preserved in our laboratory (GenBank accession number: OK019339). The screening and construction of the plasmid pET30a-AsA were previously reported [20]. The expression strain *E. coli* BL21 (DE3) was purchased from Solarbio (Beijing, China). Mut Express^®^ II Fast Mutagenesis Kit V2 and FastPure Gel DNA Extraction Mini Kit for mutant construction were purchased from Vazyme Biotech (Nanjing, China). The primers were synthesized by GenScript (Nanjing, China). Kanamycin and isopropyl-β-D-thiogalactopyranoside (IPTG) were purchased from Solarbio (Beijing, China). Modified Bradford Protein Assay Kit was purchased from Sangon Biotech (Shanghai, China). SYPRO^®^ Orange Protein Gel Stain was purchased from Sigma-Aldrich (St. Louis, USA). Enzyme substrates and all other reagents used in this research were purchased from Aladdin (Shanghai, China).

### 4.2. Site-Directed Saturation Mutagenesis

The AsA mutants were generated using QuikChange™ site-directed mutagenesis method [42] using plasmid pET30a-AsA as the template. All primers used, which contain NNK/MNN (K = G/T, M = A/C, N = A/C/G/T) degeneracy at target sites, are listed in Supplemental Table S2. The mutant plasmids were transferred into competent *E. coli* BL21 (DE3) and inoculated on the solid Luria Broth (LB) medium containing 100 μg/mL of kanamycin at 37 °C overnight. Subsequently, the grown colonies were inoculated into 96-well plates (500 μL of LB medium containing 100 mg/mL of kanamycin) and incubated at 37 °C overnight. The fermentation broth was transferred to new 96-well plates (500 mL of LB medium containing 100 mg/mL of kanamycin) with an inoculation of 5%. The transfer operation was repeated to decrease the bias between colonies. When the OD_600_ of the fermentation reached 0.6–0.8, the expression of L-asparaginases was induced by adding 100 μg/mL of IPTG and cultured at 16 °C for 20 h. The cells were harvested by centrifugation and dissolved in 50 μL of PBS (50 mM, pH 8.0). After repeated freezing at −70 °C and thawing at 30 °C, suspensions were re-centrifuged to obtain the supernatant with AsA mutant enzymes. The activities of crude AsA mutant enzymes and their residual activities after heat treatment were determined. The mutants with enhanced thermostability were sequenced and characterized.

### 4.3. Enzyme Expression and Purification

The positive mutants were transferred from the 96-well plates to the flasks for overexpression. After 16 h of induction, cells were collected by centrifugation, reconstituted with PBS (50 mM, pH 8.0), and ultrasonicated for protein extraction. Crude enzymes were obtained by centrifugation and further purified by nickel-affinity chromatography. SDS-PAGE analysis was performed on a 12% running gel to test the purity and the molecular mass of the target protein.

### 4.4. Activity Assay

The activity of L-asparaginase was established according to traditional Nessler’s method [20]. The activity assay system (1 mL) consists of 100 μL of enzyme solution (suitably diluted), 100 μL of L-asparagine (189 mM), and 700 μL of PBS buffer (50 mM, pH 8.0). After 10 min incubation at 37 °C, 100 μL of 25% trichloroacetic acid solution was added to terminate the reaction. Then, 40 μL of reaction products were added into the mix of 100 μL of Nessler’s reagent and 860 μL of ddH_2_O and measured at 436 nm to determine the amount of liberated ammonia. One international unit (IU) of L-asparaginase activity was defined as the amount of enzyme required to release 1 μM ammonia per minute under assay conditions (37 °C, pH 8.0).

### 4.5. Thermostability Analysis

The activity of the enzymes was measured at the temperature ranging from 25 to 70 °C to determine their optimum temperature, and the activity at optimum temperature for each enzyme was set as 100%, respectively. The enzymes were incubated at 40 °C (optimal temperature of AsA) to 55 °C (highest temperature at which the half-life of AsA mutants can be measured) to determine the thermostability. The residual activity of enzymes was measured at each time point with activity of unheated samples set as 100%. The half-life (*t*_1/2_) of the enzymes was calculated by fitting experimental data to the equation lnY = −kdX + b, assuming first-order inactivation (*t*_1/2_ = ln2/kd). The *T*_m_ values of the enzymes were determined using the Protein Thermal Shift assay with Applied Biosystems StepOnePlus™ qPCR instrument [43]. The reaction system was composed of 2 μL of SYPRO Orange Protein Gel Stain (100× diluted from 5000× stock solution dissolved in DMSO) and 1.0 μg of protein and subsidized up to 20 μL with PBS buffer (50 mM, pH 8.0). The qPCR program was set as follows: the temperature of the samples increased from 25 to 99 °C at a rate of 1 °C per minute, and the fluorescence intensity was measured every 1 °C.

### 4.6. Enzymatic Kinetic Analysis

The activities of mutant enzymes were determined with different L-asparagine concentrations (3.0–11.0 mM) at pH 8.0 at 37 °C, with the using enzyme concentration adjusted to make the absorbance value of reactant detectable. The Michaelis–Menten constant (*K*m), maximal reaction rate (Vmax), turnover number (*k*cat), and catalytic efficiency (*k*cat/*K*m) were calculated by the Michaelis–Menten function in the non-linear curve analysis of Origin (OriginLab, Northampton, MA, USA).

### 4.7. Consensus Analysis

The determination of homological sequences and frequency calculation of the cysteine residues were carried out by Consensus Finder Web tool [44] (http://kazlab.umn.edu, accessed on 12 November 2020), and the amino acid distributions at the corresponding sites were visualized using WebLogo 3 [45].

### 4.8. Structure Modeling

The structure model of AsA was created by SWISS-MODEL online server (https://swissmodel.expasy.org/, accessed on 29 July 2018) using *Thermococcus kodakarensis* L-asparaginase (PDB ID: 5OT0, 24.44% identity, 2.18 Å resolution) as template. The structure models of mutants were created on the base of the structure model of AsA using Coot [46]. Structure quality analysis of the models was carried out by PSVS server (http://psvs.nesg.org/, accessed on 14 April 2021). The molecular tertiary structure was visualized and analyzed in PyMOL. The enzyme interaction was calculated by the Protein Interactions Calculator (PIC) server (http://pic.mbu.iisc.ernet.in/, accessed on 6 April 2021). The potential disulfide bond in AsA was predicted by PredictProtein online server (https://predictprotein.org/, accessed on 11 November 2020).

### 4.9. MD Simulation

To analyze the contribution of the mutation to the thermostability of AsA, MD simulation of AsA wild-type and mutant C8Y/C283Q were performed. GROMACS was used for MD simulations with the Amber ff99SB force field. The AsA model was placed in a water box (SPC water model), and the distance between any protein atom and the edge of the box was set at more than 12 Å. Subsequently, 15 sodium atoms were added to the system to achieve charge balance. The system energy was minimized to a maximum force of less than 100 kJ·mol^−1^ nm^−1^ on any atom using the steepest descent integrator (2000 steps) and conjugate gradient algorithm (3000 steps). The system was equilibrated under a constant volume (NVT) ensemble and a constant pressure (NPT) ensemble for 50 ps. The final MD simulations were performed at 300 and 330 K for 50 ns. Trajectory analyses including root mean square deviation (RMSD), root mean square fluctuation (RMSF), and hydrogen bond number were achieved by using the auxiliary programs in the GROMACS package. After mutation and screening, the structure model of the effective mutants was created and analyzed by MD simulations.

### 4.10. Acrylamide Inhibition Analysis

#### 4.10.1. Potato Chips Preparation

Fresh potatoes were washed, peeled, and cut into 2 mm slices, and the starch granules on the surface of the slices were removed with ultrapure water. Potato slices were soaked in ultrapure water as control group, and soaked in enzyme solution with a final concentration of 30 IU/mL as treatment group at 37 °C for 1 h. The surface moisture of potato chips was dried out in an oven at 65 °C and potato slices were fried at 160 °C for 10 min.

#### 4.10.2. Acrylamide Extraction and Detection

Acrylamide extraction was performed according to Zhang et al. [11]. In total, a 2 g pulverized sample was mixed with 10 mL ultrapure water, ultrasonicated for 30 min, and centrifuged at 5000× *g* for 10 min. Oil in the supernatants was removed by adding hexane and centrifuging at 5000× *g* for 10 min. The aqueous layer was further purified using solid phase extraction and reconstituted with 0.5 mL of ultrapure water, and subsequently passed through 0.22 μm microporous membranes for analysis.

Acrylamide content determination was performed using liquid chromatography–tandem mass spectrometry (LC–MS/MS) with the Eclipse Plus C18 column (1.8 μm, 2.1 × 100 mm). The mobile phase consisted of methanol and water at a ratio of 5:95 with a flow rate of 0.2 mL/min, the detection wavelength was 210 nm, and the column temperature was 30 °C. The detected ions for acrylamide were m/z 72.0, 55.0, 44.1, 27.1, and standard curve of the concentration of acrylamide is shown in Figure S4.

### 4.11. Statistical Analysis

In catalytic activity and thermostability determinations of enzymes, data were expressed as mean values (±SD) of three independent experiments. In melting temperature measurement of enzymes, data were expressed as mean values (±SD) of four independent experiments. Significant difference analysis was performed using Tukey’s test (*p* < 0.05) of one-way ANOVA in GraphPad Prism 6 software (Dotmatics, San Diego, CA, USA).

## 5. Conclusions

In this study, cysteine substitution based on the consensus design was carried out to enhance the thermostability of AsA. The mutant C8Y/C283Q was obtained with a half-life of 361.64 min at 40 °C, which is over 30-fold higher than that of the wild-type, and its *T*_m_ reached 62.3 °C. Homology modeling and MD simulations were performed to explore the molecular characteristics of AsA wild-type and mutant C8Y/C283Q. The results indicated that residue Tyr8 of the mutant C8Y/C283Q was speculated to increase the thermostability by forming new aromatic interactions with other aromatic amino acids nearby. The existence of the large aromatic side chain of Tyr8 may hinder the loop to close the substrate pocket, which may be the reason for the decreased catalytic efficiency. On the other hand, residue Gln283 enhances the rigidity of the enzyme mainly via the new hydrogen bonds formed with solvent water. Compared to the AsA wild-type, the inhibition of acrylamide in fried potato chips treated with mutant C8Y/C283Q was also improved. In summary, our research provides insights into the mechanism of the improvement of thermostability of L-asparaginase and provides a reference to explore the thermostability modification of the aminohydrolases.

## Figures and Tables

**Table 1:** Activities and half-lives of cysteine residue mutants of AsA.

Mutants	Activities (IU/mL)	Residual Activities after Thermal Treatment (IU/mL)	Half-Life (45 °C, min)
Wild-type	10.4 ± 0.5 ^d^	0.0 ± 0.0 ^g^	4.1
C8V	22.6 ± 1.4 ^a^	12.3 ± 1.9 ^ef^	3.3
C8Y	20.7 ± 1.3 ^a^	72.6 ± 0.5 ^a^	57.3
C8F	17.7 ± 0.3 ^b^	34.1 ± 2.1 ^c^	27.6
C8L	13.7 ± 0.2 ^c^	21.8 ± 1.1 ^d^	3.6
C8W	16.6 ± 0.7 ^b^	22.2 ± 0.5 ^d^	12.3
C283R	13.8 ± 0.6 ^c^	16.3 ± 0.9 ^e^	3.6
C283Q	11.7 ± 0.4 ^cd^	33.0 ± 2.7 ^c^	12.5
C283D	11.1 ± 0.6 ^d^	8.6 ± 0.4 ^f^	2.0
C283L	12.8 ± 1.1 ^cd^	15.9 ± 1.4 ^e^	2.6
C283T	11.5 ± 1.4 ^cd^	8.8 ± 0.4 ^f^	2.9
C283E	11.0 ± 0.4 ^d^	44.7 ± 2.8 ^b^	4.0
C283S	12.1 ± 0.6 ^cd^	31.2 ± 1.2 ^c^	3.7

Data are expressed as mean values (±SD) of three independent experiments and different letters represent significant differences between samples (*p* < 0.05).

**Table 2:** Half-lives and *T*_m_ values of mutants.

Mutants	Half-Life (min)				*T*_m_ (°C)
	40 °C	45 °C	50 °C	55 °C	
Wild-type	9.1	4.1	ND	ND	55.2 ± 0.5 ^c^
C8Y	151.2	57.3	16.8	1.7	59.4 ± 0.8 ^b^
C8F	111.1	27.6	6.5	ND	58.2 ± 0.6 ^b^
C8W	25.2	12.3	ND	ND	57.8 ± 0.5 ^b^
C283Q	52.5	12.5	4.0	ND	59.4 ± 1.2 ^b^
C8Y/C283Q	361.6	68.0	25.2	2.5	62.3 ± 0.5 ^a^
C8F/C283Q	145.9	33.0	7.7	ND	59.6 ± 0.3 ^b^
C8W/C283Q	83.5	22.9	6.5	ND	60.8 ± 0.5 ^ab^

ND: Not determined. Data are expressed as mean values (±SD) of three independent experiments and different letters represent significant differences between samples (*p* < 0.05).

**Table 3:** Specific activities and kinetic parameters of AsA and its mutants.

Mutants	Specific Activity (IU/mg)	*K*m (mM)	Vmax (μM·min^−1^·mg^−1^)	*k*cat (s^−1^)	*k*cat/*K*m (s^−1^·mM^−1^)
Wild-type	400.8 ± 8.2 ^c^	3.8 ± 0.5 ^c^	327.7 ± 17.3 ^e^	198.0	52.0
C8Y	625.7 ± 12.1 ^a^	22.6 ± 5.0 ^a^	1663.9 ± 65.3 ^a^	1005.0	44.5
C8F	453.6 ± 41.4 ^c^	10.1 ± 2.2 ^bc^	864.7 ± 13.0 ^c^	522.3	51.8
C8W	144.3 ± 16.7 ^a^	3.3 ± 0.3 ^c^	244.3 ± 8.9 ^e^	147.6	44.7
C283Q	570.2 ± 16.7 ^b^	14.7 ± 2.8 ^b^	1165.9 ± 39.7 ^b^	704.2	48.1
C8Y/C283Q	620.8 ± 20.7 ^ab^	13.6 ± 2.0 ^bc^	1150.1 ± 104.0 ^b^	694.7	51.1
C8F/C283Q	287.5 ± 6.6 ^d^	7.4 ± 0.9 ^c^	749.3 ± 42.9 ^cd^	452.6	60.9
C8W/C283Q	217.5 ± 7.8 ^e^	6.4 ± 0.9 ^c^	648.5 ± 43.9 ^d^	391.7	61.0

Data are expressed as mean values (±SD) of three independent experiments and different letters represent significant differences between samples (*p* < 0.05).

**Table 4:** Hydrogen bonds number, Rg and SASA values of wild-type and mutant C8Y/C283Q.

Mutants	Total Hydrogen Bonds Number inside the Protein		Rg (nm)		SASA (Å^2^)	
	at 300 K	at 330 K	at 300 K	at 330 K	at 300 K	at 330 K
Wild-type	457.1 ± 10.6	461.8 ± 11.2	3.1 ± 0.9	2.6 ± 0.2	263.4 ± 4.0	260.0 ± 3.5
C8Y/C283Q	489.6 ± 10.0	494.8 ± 11.7	2.7 ± 0.4	2.7 ± 0.5	259.5 ± 3.1	256.8 ± 3.5

**Table 5:** Potential forming hydrogen bond numbers at mutated sites.

Mutants	Residues	Predicted Hydrogen Bonds Number inside the Protein		Predicted Hydrogen Bonds Number with Other Molecules	
		at 300 K	at 330 K	at 300 K	at 330 K
Wild-type	Cys8	3.4	3.0	0.0	0.6
	Cys283	1.8	1.6	2.0	1.8
C8Y/C283Q	Tyr8	5.2	5.0	0.7	1.2
	Gln283	2.3	2.2	8.6	8.3

**Table 6:** Acrylamide contents in potato slice samples after treatment with AsA wild-type and mutant C8Y/C283Q.

Mutants	Acrylamide Content (mg/kg)	Acrylamide Inhibition Rate (%)
Control	1.6559 ± 0.0630 ^a^	-
Wild-type	0.6781 ± 0.0097 ^b^	59.05 ± 3.63 ^b^
C8Y/C283Q	0.2235 ± 0.0109 ^c^	86.50 ± 0.66 ^a^

Data are expressed as mean values (±SD) of three independent experiments and different letters represent significant differences between samples (*p* < 0.05).

## Data Availability

Supplementary Materials File is available.

## Supplementary Materials

The following supporting information can be downloaded at: https://www.mdpi.com/article/10.3390/molecules27196670/s1, Table S1: Structure quality factors for AsA and mutant C8Y/C283Q models; Table S2: Oligonucleotide primers used in saturated mutation of Cys8 and Cys283; Figure S1: Alignment between AsA amino acid sequence and its consensus sequence obtained by multiple sequences alignment; Figure S2: SDS-PAGE analysis of purified mutants; Figure S3: Effect of temperature on the stability of mutant C8Y/C283Q after incubation at 45 °C for 3.5 h; Figure S4: Standard curve of the concentration of acrylamide.
