# Supplementary material for: Thermostability Improvement of L-Asparaginase from Acinetobacter soli via Consensus-Designed Cysteine Residue Substitution
Source: Molecules. 2022 Oct 7;27(19):6670. doi: 10.3390/molecules27196670 (PMC9572581; doi:10.3390/molecules27196670)
Supplement: Supplementary file 1 [file molecules-27-06670-s001.zip › molecules-1940626-supplementary.pdf]

Supporting Information for

Thermostability improvement of L-asparaginase from *Acinetobacter soli* via consensus designed cysteine residues substitution

Linshu Jiao, Huibing Chi, Bingjie Xia, Zhaoxin Lu, Xiaomei Bie, Haizhen Zhao, Fengxia Lu*, Meirong Chen*

College of Food Science and Technology, Nanjing Agricultural University, Nanjing 210095, P.R. China

*Corresponding authors:

Fengxia Lu

Tel: 0086-25-84395963; E-mail: lufengxia@njau.edu.cn

Meirong Chen

Tel: 0086-13787124062; E-mail: chenmr@njau.edu.cn

Table S1 Structure quality factors for AsA and mutant C8Y/C283Q models

Structure Quality Factors	AsA			C8Y/C283Q		
	Mean score	SD	Z-score [g]	Mean score	SD	Z-score [g]
Procheck G-factor [e] (phi / psi only)	0.03	N/A	0.43	0.03	N/A	0.43
Procheck G-factor [e] (all dihedral angles)	0.14	N/A	0.83	0.14	N/A	0.83
Verify3D	0.22	0.0000	-3.85	0.22	0.0000	-3.85
ProsaII (-ve)	0.77	0.0000	0.50	0.79	0.0000	0.58
MolProbity clashscore	0.00	0.0000	1.53	0.00	0.0000	1.53
Most favoured regions	92.8%			92.8%		
Additionally allowed regions	7.2%			7.2%		
Generously allowed regions	0.0%			0.0%		
Disallowed regions	0.0%			0.0%		

Table S2 Oligonucleotide primers used in saturated mutation of Cys8 and Cys283

Primers	Sequnences (5’-3’)
C8 F	GCCTTAATTNNKATGGGTGGAACCTTTGGTTGTATTGGT
C8 R	TCCACCCATMNNAATTAAGGCAATTTTATTCATGAGCTC
C283 F	TGGGTACATNNKGCAGGCATTGCCACCAATACCACGCAT
C283 R	AATGCCTGCMNNATGTACCCAGTCACTAATGGCATAGCG
C8Y F	TTTATATGGGTGGAACCTTTGGTTGTATTGGT
C8Y R	GGTTCCACCCATATAAATTAAGGCAATTTTATTCATCGAAT
C8F F	TTTTTATGGGTGGAACCTTTGGTTGTATTGGT
C8F R	GGTTCCACCCATAAAAATTAAGGCAATTTTATTCATCGAAT
C8W F	TTCTTATGGGTGGAACCTTTGGTTGTATTGGT
C8W R	GGTTCCACCCATAAGAATTAAGGCAATTTTATTCATCGAAT
C283Q F	GTACATCAGGCAGGCATTGCCACCAATACCAC
C283Q R	ATGCCTGCCTGATGTACCCAGTCACTAATGGCATAGC

Nucleotide sequences corresponding to the mutated amino acids are underlined.

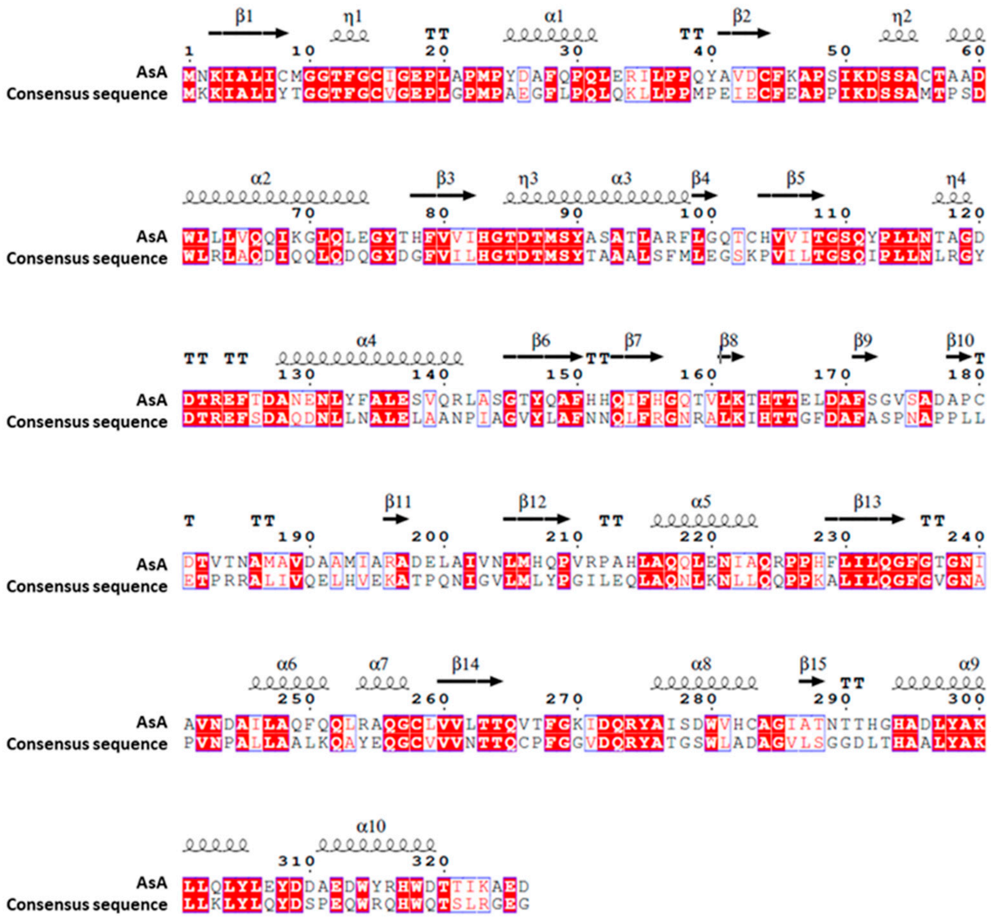

Figure S1. Alignment between AsA amino acid sequence and its consensus sequence obtained by multiple sequences alignment

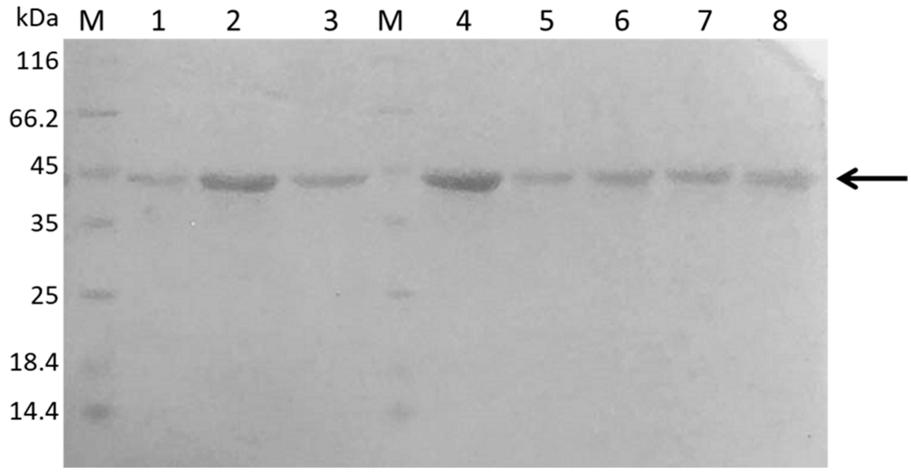

Figure S2. SDS-PAGE analysis of purified mutants at site 8 and 283

M: Protein Marker 26610; lane 1-3: mutants C8Y/C283Q, C8F/C283Q and C8W/C283Q; lane 4: AsA wild-type; lane 5-8: mutants C8Y, C8F, C8W and C283Q

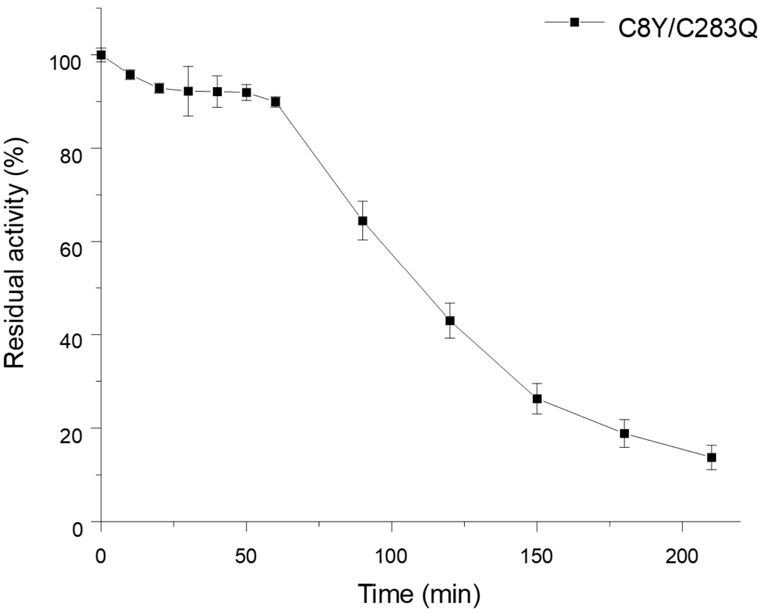

Figure S3. Effect of temperature on the stability of mutant C8Y/C283Q after incubation at 45°C for 3.5 h

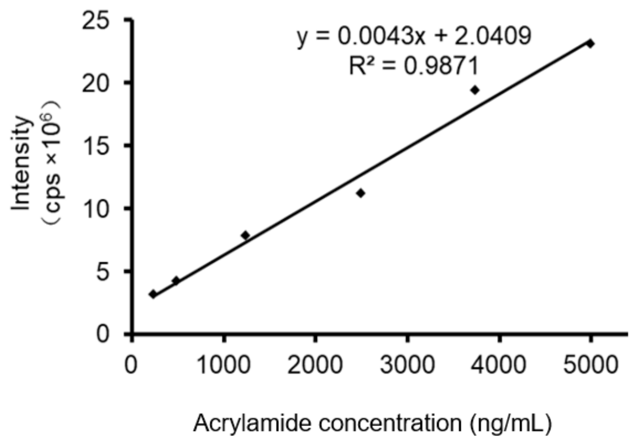

Figure S4. Standard curve of the concentration of acrylamide
